# Effects of low-dose aspirin in subjects with dyslipidemia

**DOI:** 10.1186/s12944-016-0274-8

**Published:** 2016-06-16

**Authors:** Guozhong Lou, Jianming Chen, Yu Xia

**Affiliations:** Department of Cardiology, Zhejiang Hospital, Zhejiang, China; Cardiovascular Center, Qingyuan Hospital of Traditional Chinese Medicine, Affiliated Hospital of Traditional Chinese Medicine University of Guangzhou, No.10, Qiaobei Road, Qingchen District, Qingyuan, Guangdong Province 511000 China

**Keywords:** Atherosclerotic cardiovascular diseases, Primary prevention, Safety, Efficacy

## Abstract

**Background:**

To evaluate the efficacy and safety of aspirin usage for coronary heart disease (CHD) primary prevention in patients with dyslipidemia.

**Methods:**

A cross-sectional study was conducted to enrolled subjects with documented dyslipidemia. A total of 202 patients with dyslipidemia were recruited and 138 were undergone aspirin treatment before this indexed admission and 64 had never been treated with aspirin. All subjects were undergone coronary angiography to diagnoses CHD. Clinical characteristics were collected and comparisons were performed between subjects with aspirin and subjects without aspirin therapy. Logistic regression analysis was conducted to assess the relation between aspirin and incident CHD and bleeding events.

**Results:**

Compared to those with aspirin therapy, CHD incidence was significantly higher in subjects without aspirin therapy (23.4 % versus 18.1 %, *P* < 0.05). Five patients in the aspirin group had gastrointestinal bleeding and no bleeding event was occurred in subjects without aspirin therapy. Subjects with aspirin therapy had higher rate of previous *helicobacter pylori* (HP) infection (8.7 % versus 4.7 %, *P* < 0.05). Compared to subjects without CHD, subjects with CHD were older, had higher frequencies of males and smokers, had higher heart rate, serum LDL cholesterol, Lp(a) and Hs-CRP levels. Percentages of subjects with hypertension, diabetes, gastrointestinal bleeding, and HP infection were also considerably higher in CHD group (*P* < 0.05 for all comparison). Logistic regression analysis revealed that aspirin was associated with reduced incidence of CHD, with odds ratio (OR) of 0.85 (95 % confidence interval (CI): 0.80-0.94, *P* < 0.05). Regarding safety endpoint, gastrointestinal bleeding risk associated with aspirin was attenuated to nonsignificant after adjusting for HP infection, with OR of 1.16 (95 % CI: 0.99-1.52, *P* = 0.178).

**Conclusion:**

Aspirin is beneficial for reducing incident CHD, while modestly increases gastrointestinal bleeding risk. Screening subjects with previous HP infection may avoid aspirin-related gastrointestinal bleeding.

## Background

The effects of aspirin on secondary prevention of atherosclerotic cardiovascular diseases (ASCVD) have been consistently demonstrated by previous clinical studies [1, 2]. Nonetheless, the efficacies of aspirin on ASCVD primary prevention are conflicting [3–7]. In addition, aspirin may increase bleeding risks such as incident intracranial hemorrhage and gastrointestinal bleeding [1, 8]. Therefore, it is clinically relevant and important to balance the risk and benefit of aspirin prescription for ASCVD primary prevention.

Dyslipidemia is a major risk factor of ASCVD [9], and increased serum level of low density lipoprotein (LDL) cholesterol is associated with higher incidence and prevalence of coronary heart disease (CHD) and ischemic stroke [10, 11]. Statins has been broadly used for lowering serum LDL cholesterol level, which in turn reduces cardiovascular events [10, 12, 13]. Nonetheless, whether aspirin could decrease cardiovascular risk in subjects with dyslipidemia is uncertain, and the data on the efficacy of low-dose, enteric-coated and once-daily aspirin on ASCVD primary prevention in Chinese population is also limited. Thus this, we conducted a cross-sectional research to explore whether aspirin could reduce the incidence of CHD in subjects with documented dyslipidemia. Moreover, bleeding events presumably associated with aspirin use would also be evaluated. We hoped that data from present study would shed lights for future randomized controlled trials in investigating whether aspirin prescription could confer beneficial effects on ASCVD primary prevention in subjects with dyslipidemia.

## Methods

### Studied subjects enrollment

Studied subjects were consecutively enrolled with a cross-sectional design from in-patient department and all subjects were diagnosed with dyslipidemia based on either serum lipid profiles measurement, self-report or statins treatment. Informed consent was obtained and present study was approved by participating hospital ethnic committee (GRH20150814). Study inclusion criteria were as follow: all enrolled subjects had not been diagnosed with CHD previously and were undergone coronary angiography in present indexed admission. Exclusion criteria were those currently treating with anti-coagulatory agents (e.g. warfarin) or other anti-platelet drugs (e.g. clopidogrel) were excluded. In brief, diameter of coronary artery which is equal to or more than 70 % stenosis of adjacent proximal segment is diagnosed with CHD as reviewed by two independent investigators. Studied subjects were divided into two groups in terms of with and without CHD and between-group differences were conducted. In addition, studied subjects were also divided into with and without aspirin therapy groups and between-group differences were also performed.

### Clinical characteristics collection

Demographic data including gender, age, and smoking status were extracted from in-patient medical document. Anthropometric parameters collections including systolic/diastolic blood pressure (SBP/DBP) at sitting, heart rate (HR) at rest, waist and hip circumferences using for calculating waist-hip ratio were performed by two experienced investigators. Laboratory examination results including total cholesterol (TC), triglyceride (TG), high density lipoprotein (HDL) cholesterol, LDL cholesterol, lipoprotein(a) (Lp(a)), apolipoprotein A1 (apoA1), apolipoprotein B (apoB), fasting plasma glucose (FPG) and glycated hemoglobin (HbA1c) were measured by using electrochemical method and high sensitivity C-reactive protein (Hs-CRP) was measured by using immunity transmission turbidity in our central laboratory. Medical histories including hypertension, diabetes, previous *helicobactor pylori* (HP) infection, intracranial hemorrhage or gastrointestinal bleeding presumably related to aspirin use were recorded in case report form. In brief, diagnosis of hypertension or diabetes were based on self-report, treating with anti-hypertensive or anti-diabetic medications, or measurements of indexes were higher than normal range as recommended by guideline recommendations [14, 15], respectively. Medicines use such as aspirin, statins, anti-hypertensive and anti-diabetes drugs were also recorded in case report form and underwent re-check by two independent investigators.

### Studied endpoints

The efficacy endpoints of present study were to evaluate whether aspirin could reduce the incidence of CHD in subjects with documented dyslipidemia; and the safety endpoints were to assess whether low-dose, enteric-coated and once-daily aspirin would increase bleeding risks.

### Statistical analysis

Continuous variables are presented with mean ± SD and categorical variables are presented with the number (percentages). Statistical significance of differences is analyzed using student t-test or Mann-Whitney U test for continuous variables and the chi-square or Fisher exact test for categorical variables. Logistic regression analysis was applied to calculate odds ratio (OD) and its’ associated 95 % confidence intervals (CI) of aspirin on incident CHD and bleeding events. Statistical analysis is computed using SPSS 18.0 (SPSS Inc, Chicago, IL). All of the statistical tests were two-sided and considered statistically significant if *P* < 0.05.

## Results

### Clinical characteristics of subjects with and without aspirin therapy

A total of 202 patients with documented dyslipidemia were recruited and among them, 138 had been treating with aspirin before this indexed admission and 64 had never been treated with aspirin. As shown in Table 1, male was predominant with more than 50 % of enrolled subjects, and nearly 50 % of studied subjects in both groups were smokers. No significant between-group differences in blood pressure, heart rate, lipid profiles, FPG, HbA1c and Hs-CRP were observed. Proportions of subjects with hypertension (26.1 % versus 28.1 %) and diabetes (13.8 % versus 15.6 %) were also comparable. Compared to those with aspirin therapy, CHD incidence was significantly higher in subjects without aspirin therapy (23.4 % versus 18.1 %, *P* < 0.05). Based on self-report, 5 patients in the aspirin group had gastrointestinal bleeding after regularly taking aspirin and no bleeding event occurred in subjects without aspirin therapy. Interestingly, patients in the aspirin therapy group had higher proportion of previous *helicobacter pylori* infection as self-report (8.7 % versus 4.7 %, *P* < 0.05). No significant between-group differences in medications used were observed.

**Table 1:** Comparisons between subjects with and without aspirin therapy

Variables	With aspirin (*n* = 138)	Without aspirin (*n* = 64)
Male (%)	72 (52.3)	33 (51.7)
Age (years)	53.2 ± 8.4	51.7 ± 6.9
Smoking (%)	66 (47.8)	30 (46.9)
SBP (mmHg)	135.7 ± 17.4	131.3 ± 12.8
DBP (mmHg)	71.4 ± 10.3	70.6 ± 11.6
HR (bpm)	75.5 ± 13.2	72.6 ± 13.1
Waist-Hip ratio	0.89 ± 0.03	0.88 ± 0.05
TC (mmol/L)	5.13 ± 0.24	5.11 ± 0.22
TG (mmol/L)	1.54 ± 0.32	1.57 ± 0.30
LDL (mmol/L)	3.36 ± 0.41	3.40 ± 0.36
HDL (mmol/L)	0.93 ± 0.06	0.92 ± 0.06
Lp(a) (mg/L)	238.96 ± 26.45	244.35 ± 33.15
ApoA1 (mmol/L)	1.06 ± 0.33	1.05 ± 0.29
ApoB (mmol/L)	1.10 ± 0.26	1.11 ± 0.30
FPG (mmol/L)	5.3 ± 0.6	5.4 ± 0.5
HbA1c (%)	5.9 ± 0.8	5.8 ± 0.6
Hs-CRP (mg/L)	10.7 ± 3.5	11.4 ± 3.2
Hypertension (%)	36 (26.1)	18 (28.1)
Diabetes (%)	19 (13.8)	10 (15.6)
CHD (%)	25 (18.1)*	15 (23.4)
Intracranial hemorrhage (%)	0 (0)	0 (0)
Gastrointestinal bleeding (%)	5 (3.6)*	0 (0)
HP infection (%)	12 (8.7)*	3 (4.7)
Statins (%)	127 (92.0)	60 (93.8)
Anti-hypertension (%)	27 (19.6)	11 (17.2)
Anti-diabetes (%)	15 (10.9)	8 (12.5)

Denote: * *P* < 0.05 versus without aspirin group

### Clinical characteristics of subjects with and without CHD

All 202 subjects were separated into two groups based on coronary angiography findings. As shown in Table 2, compared to subjects without CHD, subjects with CHD had more male patients, were older and had more smokers (*P* < 0.05 for all comparison). And subjects with CHD had significantly higher heart rate, serum LDL cholesterol, Lp(a) and Hs-CRP levels. Furthermore, proportions of subjects with hypertension, diabetes, gastrointestinal bleeding, HP infection and anti-hypertensive therapy were also considerably higher in CHD group compared to without CHD group (*P* < 0.05 for all comparison).

**Table 2:** Comparisons between subjects with and without CHD

Variables	CHD(*n* = 40)	Without CHD(*n* = 162)
Male (%)	23(57.5)*	82(50.6)
Age (years)	55.4 ± 8.7*	51.2 ± 7.3
Smoking (%)	22(55.0)*	74(45.7)
SBP (mmHg)	134.2 ± 14.5	132.6 ± 16.3
DBP (mmHg)	73.5 ± 10.1	71.4 ± 8.6
HR (bpm)	77.3 ± 8.0*	71.4 ± 6.6
W-H ratio	0.90 ± 0.04	0.88 ± 0.05
TC (mmol/L)	5.28 ± 0.23	5.04 ± 0.22
TG (mmol/L)	1.66 ± 0.30	1.59 ± 0.37
LDL (mmol/L)	3.49 ± 0.44*	3.20 ± 0.38
HDL (mmol/L)	0.89 ± 0.17	0.92 ± 0.20
Lp(a) (mg/L)	258.56 ± 30.86*	227.16 ± 24.43
ApoA1 (mmol/L)	1.04 ± 0.27	1.07 ± 0.22
ApoB (mmol/L)	1.15 ± 0.33	1.10 ± 0.29
FPG (mmol/L)	5.5 ± 0.5	5.2 ± 0.6
HbA1c (%)	5.9 ± 0.7	5.6 ± 0.5
Hs-CRP (mg/L)	13.4 ± 3.8*	10.7 ± 3.1
Hypertension (%)	16(40.0)*	38(23.5)
Diabetes (%)	9(22.5)*	20(12.3)
Intracranial hemorrhage (%)	0(0)	0(0)
Gastrointestinal bleeding (%)	3(7.5)*	2(1.2)
HP infection (%)	4(10.0)*	11(6.8)
Statins (%)	37(92.5)	150(92.6)
Anti-hypertension (%)	11(27.5)*	27(16.7)
Anti-diabetes (%)	5(12.5)	18(11.1)

Denote: * *P* < 0.05 versus without CHD group

### Logistic regression analysis

Logistic regression analysis was performed to evaluate the relation between aspirin use and incident CHD and gastrointestinal bleeding. As shown in Table 3, in CHD outcome model (efficacy endpoint), after adjusting for potential confounding covariates including age, gender, smoking status, SBP, LDL cholesterol, Lp(a), Hs-CRP, hypertension, diabetes and statins, aspirin therapy reduced the risk of CHD, with OR of 0.85 (95 % CI: 0.80-0.94, *P* < 0.05). In gastrointestinal bleeding outcome model (safety endpoint), after adjusting for potential confounding covariates including age, gender, smoking, LDL cholesterol, hypertension and diabetes, the risk of gastrointestinal bleeding associated with aspirin therapy remained significant (OR: 1.88 and 95 % CI: 1.56-2.33, *P* < 0.05).

**Table 3:** Odds ratio of CHD and gastrointestinal bleeding in subjects with aspirin

Outcomes	With Aspirin (*n* = 138)	OR (95 % CI)
CHD, n (%)	25 (18.1)	0.85 (0.80–0.94)*
Gastrointestinal bleeding, n (%)	5 (3.6)	1.16 (0.99–1.52)

Denote: *P < 0.05

In CHD outcome model, fully adjusting for age, gender, smoking, SBP, LDL cholesterol, Lp(a), Hs-CRP, hypertension, diabetes and statins

In Gastrointestinal bleeding model, fully adjusting for age, gender, smoking, LDL cholesterol, hypertension, diabetes and HP infection

## Discussion

Our preliminary data show that aspirin may be beneficial for decreasing incidence of CHD as documented by coronary angiography in subjects with dyslipidemia. Nonetheless, aspirin may also slightly increase gastrointestinal bleeding risk, especially in subjects with previous HP infection which deserves further investigation.

Aspirin now has been broadly used for ASCVD secondary prevention owing to its compelling evidence on improving cardiovascular outcomes [16]. Nonetheless, data from primary prevention studies are controversial. In addition, aspirin therapy may increase bleeding risk and the major mechanisms are twofold [8]. On the one hand, aspirin has effect on inhibiting cyclooxygenase thereby reducing platelet activation and adhesion. On the other hand, aspirin also has unwanted effect with respect to causing gastritis or gastric ulceration. Therefore, it should be cautious to balance the risk and benefit of prescribing aspirin before more solid evidence is available to demonstrate aspirin should be applied to subjects with ASCVD risk factors such as dyslipidemia.

We performed a cross-sectional research to investigate the relation between aspirin use and incidence of CHD as diagnosed by indexed coronary angiography. All our recruited subjects were diagnosed with dyslipidemia and more than 90 % was treated with statins. Results showed that after fully adjusting for potential confounding covariates, aspirin therapy was beneficial for reducing the incidence of CHD. However, the data from the Japanese Primary Prevention Project (JPPP) trial showed that aspirin therapy was not beneficial for reducing composite cardiovascular outcomes including nonfatal ischemic stroke, myocardial infarction and vascular death [3]. There were several differences between our present study and the JPPP trial. First of all, the differences in studied design and ethnic group might largely account for these discrepancies. Second, we only investigated the effect of aspirin on CHD incidence while the JPPP trial additionally included ischemic stroke and vascular death as efficacy endpoints. It might be possible that aspirin has different effects on coronary artery and cerebral vessels, given the heterogeneity of mechanisms underlying cerebrovascular ischemia [17]. Third, subjects in our present study were much younger than those in the JPPP trial, and aging might offset the potential benefits of aspirin therapy [18]. Moreover, only a minority of subjects in our present study had co-morbidities such as hypertension and diabetes, which indicated that subjects in our present study were at relatively lower ASCVD risk compared to those in the JPPP trial. Furthermore, in another study investigating the effect of low-dose aspirin to prevent cardiovascular events in subjects with type 2 diabetes mellitus, Hisao and colleagues reported that on cardiovascular benefits were found [5]. In a large meta-analysis, it was also revealed that aspirin for primary prevention, the cardiovascular benefit is uncertain. However, it might increase bleeding risk [1]. Likewise, different study design and populations should largely be accounted for these discrepancies. In addition, with respect to our present findings, we considered that the benefit of aspirin for ASCVD primary prevention might be possible for a specific population but not the whole general population.

We further evaluated the incidence of bleeding events presumably associated with aspirin therapy. We mainly included intracranial hemorrhage and gastrointestinal bleeding as safety endpoints because these two adverse events were accounted for a vast majority of aspirin-related bleeding complications. Based on self-report, 5 cases of gastrointestinal bleeding presumably associated with aspirin therapy were identified and after initially adjusting for potential confounding factors, aspirin therapy appeared to increase gastrointestinal bleeding risk. Nonetheless, bleeding risk associated with aspirin therapy was attenuated to nonsignificant after additionally adjusting for HP infection. As is well known that HP infection increases the incidence of gastric and duodenal ulceration [19, 20], and the prevalence of HP infection in China is much higher than the other developed countries [21]. Therefore, it was conceivable that subjects with HP infection in our current study might have occult gastrointestinal ulceration which exposed them at higher risk of gastrointestinal bleeding. These findings indicated that screening for HP infection might help reduce potential bleeding risk associated with aspirin therapy. In addition, smoking may also increase bleeding risk in relation to its effects on impairing coagulatory system although since no significant between-group difference in smoking rate was observed.

Comparisons between subjects with and without CHD revealed that subjects with CHD might at increased risk of gastrointestinal bleeding. For example, subjects with CHD were with male predominant, older, higher percentages of smoking, diabetes and HP infection, all of which were significant contributors of gastrointestinal bleeding. These findings might indicate that subjects at increased risk of CHD might also have a higher risk of gastrointestinal bleeding concomitantly.

There were several limitations of our current study. First of all, this was a cross-sectional research and the results from present study could not be used to conclude a causal relationship. Rather, present study might provide pilot data for future randomized perspective study to explore the effect of aspirin for CHD primary prevention. Second, we did not include ischemic stroke as an efficacy outcome because we did not have solid evidence to diagnose ischemic stroke in the absence of compute tomography scan. Owing to a high prevalence and incidence of ischemic stroke in Chinese population, it is clinically relevant to include ischemic stroke as an efficacy endpoint in future study. Third, the diagnoses of gastrointestinal bleeding and HP infection were based on self-report and therefore, the accuracy of the safety endpoint evaluation might be somewhat compromised. However, we had enquired both the patients’ immediate relative and charged physicians in other hospitals. Therefore, we considered that the possibility of diagnostic inaccuracy was small. Last but not the least, a small sample size of present study was not adequately powered to detect potential between-group differences.

## Conclusion

Our current study shows that in subjects with dyslipidemia, low-dose, enteric-coated, once-daily aspirin is beneficial for reducing the incidence of CHD while only slightly increase gastrointestinal bleeding risk.
